# Recent advances in the molecular mechanism of mitochondrial calcium uptake

**DOI:** 10.12688/f1000research.15723.1

**Published:** 2018-11-28

**Authors:** Giorgia Pallafacchina, Sofia Zanin, Rosario Rizzuto

**Affiliations:** 1 Department of Biomedical Sciences, University of Padua, Padua, 35131, Italy; 2 Italian National Research Council (CNR), Neuroscience Institute, Padua, 35131, Italy; 3 Department of Medicine, University of Padua, Padua, 35128, Italy

**Keywords:** Mitochondria, mitochondrial Ca2+ uptake, mitochondrial ion transport, MCU, MICU

## Abstract

In the last few decades, a large body of experimental evidence has highlighted the complex role for mitochondria in eukaryotic cells: they are not only the site of aerobic metabolism (thus providing most of the ATP supply for endergonic processes) but also a crucial checkpoint of cell death processes (both necrosis and apoptosis) and autophagy. For this purpose, mitochondria must receive and decode the wide variety of physiological and pathological stimuli impacting on the cell. The “old” notion that mitochondria possess a sophisticated machinery for accumulating and releasing Ca ^2+^, the most common and versatile second messenger of eukaryotic cells, is thus no surprise. What may be surprising is that the identification of the molecules involved in mitochondrial Ca ^2+^ transport occurred only in the last decade for both the influx (the mitochondrial Ca ^2+^ uniporter, MCU) and the efflux (the sodium calcium exchanger, NCX) pathways. In this review, we will focus on the description of the amazing molecular complexity of the MCU complex, highlighting the numerous functional implications of the tissue-specific expression of the variants of the channel pore components (MCU/MCUb) and of the associated proteins (MICU 1, 2, and 3, EMRE, and MCUR1).

## Introduction

Ca ^2+^ is universally recognised as one of the most pleiotropic second messengers in cell biology. Indeed, Ca ^2+^ ions are responsible for decoding a variety of extracellular and intracellular stimuli, which, in animals, range from endocrine secretion to gene expression, muscle contraction, and synaptic transmission ^1– 5^. The efficacy of Ca ^2+^ as a signalling molecule relies mainly on the maintenance of a steep Ca ^2+^ gradient between the concentration in the extracellular (few mM) and intracellular (∼100 nM) environments. This >10,000-fold difference ensures that even very small changes in intracellular Ca ^2+^ concentration ([Ca ^2+^] _i_) are effective in regulating the numerous Ca ^2+^-sensitive proteins of the cell, such as catalytic enzymes, channels, and transcription factors ^6^. The maintenance of a low [Ca ^2+^] _i_ is tightly controlled by the presence of pumps and transporters both at the plasma membrane ^7^ and at the membrane of organelles accumulating large amounts of Ca ^2+^ ^8, 9^. The endoplasmic/sarcoplasmic reticulum (ER or SR in striated muscle) is undoubtedly the main intracellular Ca ^2+^ store; nevertheless, other cellular compartments actively participate in modulating [Ca ^2+^] _i_: first of all mitochondria but also the Golgi apparatus ^10^, endosomes, and lysosomes ^2, 5, 11^.

The role for mitochondria in Ca ^2+^ handling was first demonstrated in the 1960’s, when their ability to actively accumulate Ca ^2+^ was evaluated in different cellular *ex vivo* models ^12– 14^. This occurred even before the advent of the chemiosmotic theory ^15^, which provided the thermodynamic basis for Ca ^2+^ entry into mitochondria. In addition, the role for mitochondria in cell function has gradually expanded, and its initial identification as the powerhouse for cellular energy supply was subsequently integrated into a more complex activity that includes the regulation of cell death, metabolism, and signalling pathways ^16– 18^. Notably, many mitochondrial functions are directly regulated by the level of Ca ^2+^ ions inside the organelles ^4, 19^. The control of mitochondrial Ca ^2+^ concentration ([Ca ^2+^] _mt_) is thus of primary relevance for cell physiology, and emerging evidence converges on the concept that its dysregulation is of utmost importance in the establishment of pathological conditions.

**Figure 1.**
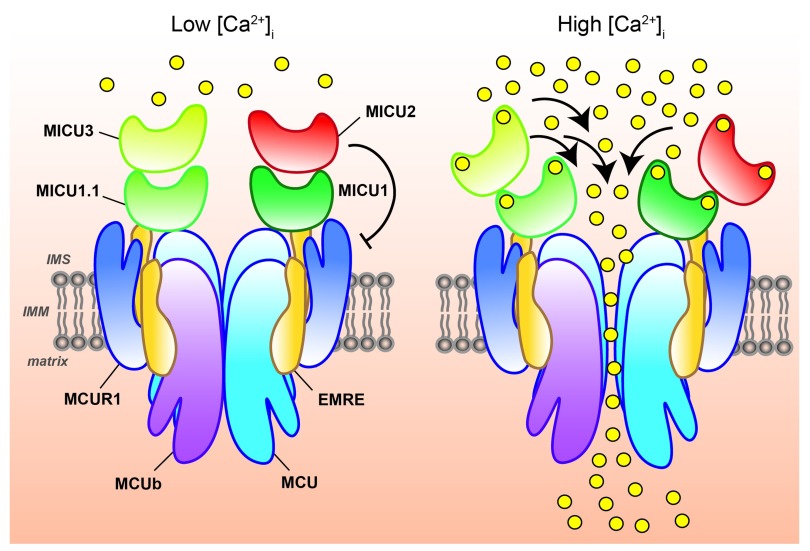
The mitochondrial calcium uniporter (MCU) complex. Schematic representation of MCU-mediated Ca ^2+^ entry into mitochondria at different intracellular Ca ^2+^ concentrations ([Ca ^2+^] _i_). Mitochondrial Ca ^2+^ uptake is controlled by a multiprotein complex consisting of MCU and MCUb (the pore-forming subunits) together with the essential mitochondrial Ca ^2+^ uniporter regulator (EMRE), the mitochondrial Ca ^2+^ uptake (MICU) proteins, MICU1, MICU1.1, MICU2, and MICU3, and, possibly, the MCU regulator 1 (MCUR1). At low [Ca ^2+^] _i_, MICU1/MICU1.1–MICU2 or MICU1/MICU1.1–MICU3 heterodimers ensure MCU gatekeeper activity, preventing undesirable mitochondrial Ca ^2+^ cycling in resting cells. At high [Ca ^2+^] _i_, the MICU proteins act as positive regulators of MCU channel activity, allowing efficient mitochondrial Ca ^2+^ uptake (right). IMS, intermembrane space; IMM, inner mitochondrial membrane.

In this commentary, we will focus on the molecular machinery underlying mitochondrial Ca ^2+^ uptake, the mitochondrial Ca ^2+^ uniporter (MCU) complex (see Figure 1), to which our laboratory has dedicated much effort in the last decade, contributing to its discovery in 2011 and unravelling its molecular complexity and functional significance.

## The mitochondrial Ca ^2+^ uniporter complex

Since the first reports of mitochondrial Ca ^2+^ uptake ^12– 14^, extensive studies have been conducted to identify the mitochondrial Ca ^2+^ entry mechanism, characterised by Ruthenium Red sensitivity, high selectivity, and high capacity for the cation ^20^. Only from 2010, with the identification of the first regulator of the mitochondrial Ca ^2+^ channel, the mitochondrial Ca ^2+^ uptake protein 1 (MICU1) ^21^, and, one year after, by the cloning and molecular characterisation of the gene coding for the MCU ^22, 23^, the entry pathway was characterised at the molecular level and the route for the genetic manipulation of mitochondrial Ca ^2+^ uptake was finally open.

### The mitochondrial Ca ^2+^ uniporter MCU

In 2011, two independent *in silico* screenings ^22, 23^ identified the *CCDC109a* gene product as the long-sought pore-forming unit of the MCU channel mediating mitochondrial Ca ^2+^ uptake in mammalian cells. Purified MCU protein reconstitution in lipid bilayers revealed a Ca ^2+^ channel activity with the electrophysiological features of the hypothetical MCU ^20^. In addition, it was clearly shown that MCU downregulation in mammalian cells inhibits mitochondrial Ca ^2+^ uptake while its overexpression increases mitochondrial Ca ^2+^ accumulation, thus proving that it is *bona fide* the channel mediating Ca ^2+^ uptake in mitochondria ^23^.

The sequence of MCU consists of two transmembrane α-helix domains spanning the inner mitochondrial membrane (IMM), the second of which contains the critical DIME motif responsible for the channel selectivity filter, linked by a short loop toward the mitochondrial intermembrane space (IMS), while both the C- and the N-termini face the mitochondrial matrix ^22, 24^. A single MCU molecule *per se* is thus not able to form the channel but should organise in oligomers, and MCU was initially proposed to arrange itself into tetramers ^25^. This hypothesis was very recently confirmed by coherent high-resolution cryo-EM data from four independent groups on different MCU orthologs ^24, 26– 28^. These seminal studies finally unveiled the structure and arrangement of MCU protomers within the complex and the exact position of the channel selectivity filter. The 3D reconstruction of EM data unambiguously revealed that MCU forms tetramers with a nonobvious symmetry: the transmembrane domain displays a fourfold symmetry, while the N-terminal domain (NTD) on the matrix side shows a twofold symmetry axis. These findings confuted the pentameric MCU architecture that was previously proposed based on magnetic resonance and negative staining EM data from *Caenorhabditis elegans* MCU-ΔNTD protein, in which a significant portion of the NTD was removed to facilitate the analysis ^29^. In addition, the MCU DIME motif, which contains the two critical acidic residues directly involved in the cation coordination, has now been shown to reside at the beginning of––and thus integral to––the second transmembrane helix (TMH), and not in the loop connecting the two transmembrane helices, as previously suggested ^29^.

However, as Raffaello *et al*. showed, MCU is not the only pore-forming unit of the oligomer, but it associates with the protein encoded by the MCU paralog gene *CCDC109b,* which was thus named MCUb ^25^. The characterisation of MCUb demonstrated that it acts as a negative regulator of MCU activity both in lipid bilayer experiments and when overexpressed in mammalian cells ^25^ (see Figure 1). To note, in other organisms, such as *Trypanosoma cruzi*, the ortholog of MCUb acts as a Ca ^2+^ conducting subunit and its overexpression enhances, rather than dampens, mitochondrial Ca ^2+^ uptake ^30^. Interestingly, in another trypanosomatid, *Trypanosoma brucei*, two additional MCU isoforms were identified, MCUc and MCUd, which are also endowed with Ca ^2+^ uniporter activity and are able to form heterotetrameric complexes with their homologues MCU and MCUb ^31^. This highlights the existence of significant species-specific differences in the function and distribution of MCU orthologues, despite their sequence conservation, and this should be taken into account when studying different organisms. Moreover, the expression and relative proportion of MCU and MCUb vary significantly among different tissues in mammals; thus, each cell type may have different variants of the uniporter owing to the specific composition and stoichiometry of MCU subunits. This accounts for tissue-specific variations of the capacity of mitochondria to take up Ca ^2+^ and perfectly fits with the electrophysiological recordings of MCU Ca ^2+^ currents in mitochondria from different mammalian tissues ^32^. In skeletal muscle, for example, the presence of a high MCU:MCUb ratio ^25^ matches the highest mitochondrial Ca ^2+^ conductance recorded in this tissue ^32^, whereas, in adult heart, the relatively elevated MCUb expression ^25^ results in a considerably low Ca ^2+^ current in cardiomyocyte mitochondria ^32^. In cardiac cells, in which ∼37% of the volume is occupied by mitochondria ^33^, this is crucial for preventing massive mitochondrial Ca ^2+^ accumulation that would potentially cause undesired buffering of the [Ca ^2+^] _i_ transients required for contraction, futile cycling of Ca ^2+^ across the IMM, and, eventually, organelle Ca ^2+^ overload and apoptosis. The control of the MCU:MCUb proportion in the mitochondrial Ca ^2+^ channel pore domain is thus fundamental for the function and physiology of different tissues.

After the molecular identification of the MCU pore components and auxiliary factors, the path for the genetic manipulation of mitochondrial Ca ^2+^ uptake machinery in cells and animal models was finally opened. The first *MCU ^–/–^* mouse was generated by Finkel’s group in 2013. It shows a relatively mild phenotype, and, despite the expected abrogation of mitochondrial Ca ^2+^ uptake, it develops normally and displays unaffected basal metabolism ^34^. However, the *MCU ^–/–^* mouse shows increased plasma lactate levels after starvation and impaired exercise performance accompanied by a reduction in the activity of pyruvate dehydrogenase in skeletal muscle ^34^. These findings, together with the fact that *MCU ^–/–^* mouse survival depends on the genetic background ( *MCU* deletion is embryonically lethal in a pure C57/BL6 background ^35^), pointed to a more subtle and less obvious involvement of mitochondrial Ca ^2+^ uptake in organ metabolism and organism development. This issue has been elegantly addressed by recent studies implementing tissue-specific modulation of MCU expression (by ablation/downregulation or overexpression) in adult tissues. For example, cardiac-specific tamoxifen-inducible *MCU* deletion in adult mice, differently from the germline genetic ablation of *MCU*, which did not protect from ischemic-reperfusion injury ^34^, clearly protects from ischemia-reperfusion heart damage, abrogates the contractile responsiveness to β-adrenergic stimulation responsible for the so-called “fight-or-flight” response, and reduces heart bioenergetics reserve capacity, even though it does not induce phenotypic abnormalities either in basal conditions or after cardiac overload and does not alter cardiomyocytes’ resting [Ca ^2+^] _mt_^36– 39^. These results suggest that MCU may be dispensable for cardiac homeostasis in basal conditions, while it appears to play a major role in cardiac metabolic flexibility during acute stress. Similarly to what has been reported for the heart tissue, germline deletion of *MCU* did not protect from hypoxic/ischemic brain injury ^40^. However, brains from conditional neuronal-specific inducible *MCU*-deleted mice as well as primary cortical neurons silenced for *MCU* show a significant reduction of the hypoxic/ischemic damage and decreased cell death without impairment of neuronal mitochondria metabolism ^41^. Once more, these findings point to the existence of a strong drive for organs and tissues to respond to chronic *MCU* ablation by establishing adaptive metabolic shunts in order to cope with/bypass impaired mitochondrial Ca ^2+^ signalling. Such adaptation to mitochondria dysfunction has also been described in the cardiac-specific *Tfam ^–/–^* mouse, the murine model for human cardiomyopathies with mtDNA depletion ^42^, in which the alteration in mitochondrial metabolism due to oxidative phosphorylation (OXPHOS) deficiency induces a secondary upregulation of MCU protein and a concomitant downregulation of *NCLX* transcription ^43^. The resulting increased mitochondrial Ca ^2+^ uptake and reduced Ca ^2+^ efflux in *Tfam ^–/–^* cardiac mitochondria, although inducing an elevated and potentially damaging [Ca ^2+^] _mt_, appear somehow functional to ensure an efficient Ca ^2+^-dependent mitochondrial respiration that otherwise would be compromised by the pathology ^41^.

Overall, these findings lead us to the following consideration: experimental systems featuring long-term genetic manipulation of the mitochondrial Ca ^2+^ uptake machinery should be evaluated with caution, and the potential induction of unpredicted phenotypes, as a consequence of adaptive/maladaptive responses, should be taken into account. This is particularly relevant when *in vivo* models are considered.

**Figure 2.**
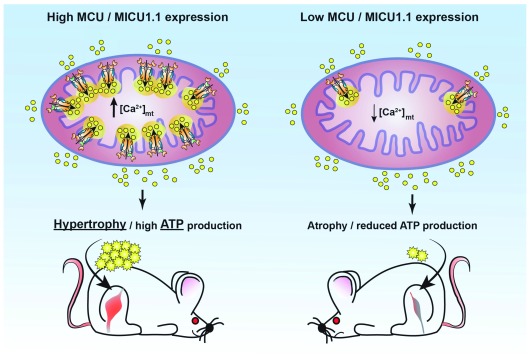
The role of mitochondrial Ca ^2+^ signalling in skeletal muscle trophism. Schematic representation of the effects of manipulating mitochondrial Ca ^2+^ uptake in skeletal muscle fibres. Increased mitochondrial Ca ^2+^ accumulation by enhanced expression of mitochondrial Ca ^2+^ uniporter (MCU) or mitochondrial Ca ^2+^ uptake protein 1.1 (MICU1.1) leads to muscle hypertrophy by stimulating fibre growth and ATP production (left). Reduction of mitochondrial Ca ^2+^ uptake by downregulation of either MCU or MICU1.1 causes fibre atrophy and impaired ATP production (right). [Ca ^2+^] _mt_, mitochondrial Ca ^2+^ concentration.

In line with this, the acute but transient manipulation of MCU expression in skeletal muscle provided additional intriguing evidence of the wide-ranging influence of mitochondrial Ca ^2+^ signalling in the maintenance of organ and organism homeostasis. The decrease or enhancement of [Ca ^2+^] _mt_ by AAV-mediated MCU silencing or overexpression, respectively, demonstrated that mitochondrial Ca ^2+^ uptake regulates myofibre trophism through the modulation of the activity of the insulin growth factor-1/Akt pathway and the transcription of the peroxisome proliferator-activated receptor gamma coactivator 1 alpha 4 gene ( *PGC1α4*). In particular, the overexpression of MCU in both adult and neonatal muscles induces significant fibre hypertrophy, which is not accompanied by alterations in mitochondrial membrane potential or other metabolic parameters (such as glycogen content and succinate dehydrogenase activity) ^44^. On the contrary, MCU downregulation leads to marked fibre atrophy as demonstrated by reduction of fibre size, inhibition of Akt activity, reduction of *PGC1α4* transcripts, and impaired activation of the pyruvate dehydrogenase complex ^44^, in agreement with the data from the *MCU ^–/–^* mice ^34^ (see Figure 2).

Moreover, the recent description of a mouse model bearing constitutive skeletal muscle-specific deletion of MCU ( *skMCU ^–/–^* mice) has been fundamental to uncover the metabolic rewiring occurring at the level of skeletal muscle cell but also, importantly, systemically ^45^. In line with previous data ^34, 44^, the muscle of *skMCU ^–/–^* mice shows decreased performance and smaller fibre size compared to that of control mice ^45^. Moreover, it shows a shift toward fast fibre type and an enhanced preference for fatty acid oxidative metabolism ^45^. Interestingly, the ablation of *MCU* in skeletal muscle also impinges on other tissues, as shown by the increased catabolic response in both liver and adipose tissue of *skMCU ^–/–^* animals ^45^. This clearly points out how the mitochondrial metabolic adaptation in response to altered Ca ^2+^ dynamics (i.e. *MCU* deletion) within the muscle tissue may have major systemic impact on different tissues and, importantly, on the modulation of global metabolism.

### The MICU proteins

MICU1 was the first regulator of mitochondrial Ca ^2+^ uptake to be identified ^21^, even before the identification of MCU itself. It was initially described as an EF-hand Ca ^2+^-binding protein associated with the IMM that stimulates MCU channel opening and thus acts as a positive modulator of mitochondrial Ca ^2+^ uptake. Subsequent findings defined the crucial role of MICU1 in keeping the channel inactive under resting conditions, i.e. at low [Ca ^2+^] _i_^46, 47^, thus preventing massive Ca ^2+^ entry that will otherwise cross the IMM down the steep electrochemical gradient and cause harmful mitochondrial Ca ^2+^ overload. MICU1 was thus appropriately defined as the gatekeeper of the MCU channel. This concept has been recently confirmed by two independent *in vivo MICU1 ^–/–^* mouse models. These *MICU1 ^–/–^* animals, despite normal embryonic development and growth, show a variable degree of postnatal lethality (one out of six to one out of seven surviving pups in the case of CRISPR/Cas9 mutants ^48^ and 100% lethality in the case of Cre recombinase/LoxP deleted alleles ^49^) mostly due to breathing failure for the underdevelopment of the respiration coordination centres (cerebellum and Purkinje cells) ^49^. The few surviving knockout mice display muscle impairment and ataxia ^48^, a phenotype very similar to that described in children with a *MICU1* mutation ^50^. The analysis of *MICU1 ^–/–^* mitochondria revealed increased matrix [Ca ^2+^] _mt_, increased sensitivity to permeability transition pore (PTP) opening and cell death, and reduced production of ATP, similar to what was reported in *MICU1*-silenced hepatocytes and Hela cells ^46, 47^. Overall, these data clearly highlight the relevance of MICU1 gatekeeping function in the control of mitochondrial Ca ^2+^ homeostasis and metabolism. Nevertheless, an additional intriguing feature of MICU1 action has recently emerged, which consists of its ability to confer MCU selectivity for Ca ^2+^ over Mn ^2+^. Indeed, the DIME motif in the MCU pore subunit appears to be unable *per se* to discriminate between Ca ^2+^ and Mn ^2+^ in the absence of MICU1 ^51^. However, when present, MICU1 prevents Mn ^2+^ entry in mitochondria, thus ensuring the stringent Ca ^2+^ selectivity of MCU ^51^. In this scenario, the proper stoichiometry of MICU1 is thus crucial to avoid mitochondrial Mn ^2+^ uptake and to prevent Mn ^2+^ toxicity. This concept may have relevant implications for human pathologies, especially for those diseases caused by MICU1 deficiency ^50, 52, 53^ but also for Parkinson’s disease, as already suggested ^51^.

Additional MICU family members have been identified by genome sequence analysis soon after MICU1, namely MICU2 (EFHA1) and MICU3 (EFHA2) ^54^, which share 41% and 34% identity with MICU1, respectively, but display a broad and non-overlapping expression pattern compared to MICU1 ^55^. In particular, MICU2 was shown to bind covalently to MICU1 through disulphide bridges, and the resulting dimer endows the genuine MCU gatekeeper activity ^56^. In this scenario, cytosolic Ca ^2+^ elevation causes Ca ^2+^ binding to the EF-hand domains of the MICU1–MICU2 dimer, allowing MICU1 to function as a positive regulator of MCU activity, thus efficiently promoting Ca ^2+^ entry into the organelle ^56^ (see Figure 1).

As for MICU3, its evolutionary conservation and sequence similarity to MICU1 suggested that it may share a common biological function with MICU1 ^54^. Along this line, its role as a positive regulator of mitochondrial Ca ^2+^ uptake has been demonstrated recently ^55^. Indeed, the expression of MICU3 in Hela cells, which normally do not express it, showed that it interacts with MICU1, but not with MICU2, forming obligatory dimers ^55^ (see Figure 1). This interaction causes a significant increase of mitochondrial Ca ^2+^ uptake, demonstrating the stimulatory action of MICU3 on MCU activity. In addition, while displaying a reduced gatekeeper activity, MICU3 has been shown to mediate a more rapid response of mitochondria to [Ca ^2+^] _i_ changes, allowing a shorter delay between the [Ca ^2+^] _i_ rise and the increase in mitochondria Ca ^2+^ uptake compared to MICU1 ^55^. MICU3 thus plays a pivotal role in determining the kinetics of mitochondrial Ca ^2+^ signalling and consequently in the modulation of global [Ca ^2+^] _i_ dynamics, which may be of utmost relevance for the decoding of Ca ^2+^ signals in excitable cells, such as neurons, where indeed MICU3 shows the highest level of expression ^54, 55^.

By the interaction of monomers with non-overlapping functions, the MICU proteins confer an important property of the mitochondrial Ca ^2+^ uptake system: the sigmoidicity of the dose-response to Ca ^2+^. Indeed, the activity of MCU, which is kept low at resting [Ca ^2+^] _i_ (∼100 nM) owing to the MICU1–MICU2 gatekeeper action, as already discussed, becomes extremely efficient upon [Ca ^2+^] _i_ elevation. Indeed, Ca ^2+^ binding to the MICU1–MICU2 regulatory complex converts it from an inhibitory regulator (ensuring gatekeeping) at basal [Ca ^2+^] _i_ to an activator, strongly enhancing the flux through the channel. The threshold for MCU activation is directly dependent on the Ca ^2+^ affinity of MICUs’ EF hands (which range from 300 nM to 1.3 µM), since the cooperative Ca ^2+^ binding to these domains induces a conformational change in the MICU1–MICU2 dimer, which acts as a molecular switch relieving MCU inhibition ^57^.

Despite the role for MICU2 in the regulation of MCU gatekeeping and activation being controversial still, an intriguing hypothesis is that it has evolved to spatially restrict the Ca ^2+^ crosstalk between single inositol trisphosphate receptor (IP3R) and MCU channels ^58^. Indeed, the presence of MICU2 has been shown to decrease the Ca ^2+ ^affinity of the MICU1 gatekeeper, thus elevating the threshold for MCU opening ^58^. In the context of the intracellular environment, this increased threshold for mitochondrial Ca ^2+^ uptake translates into the need for a shorter distance between the ER Ca ^2+^-releasing channel (IP3R) and the mitochondrial Ca ^2+^ uptake channel (MCU) ^58^.

In line with this, in the last few years, the stoichiometry of MCU components has emerged as a crucial feature of the modulation of mitochondrial Ca ^2+^ uptake rate. The relative proportion of the MICU proteins, in particular, has been demonstrated as the basis of the tissue-specific differences in cellular Ca ^2+^ dynamics ^59^. Indeed, the different ratio of MICU1:MCU and MICU1:MICU2 proteins in the liver, the heart, and skeletal muscle has been correlated with the distinct mitochondrial Ca ^2+^ handling of these three tissues ^59^. The level of MICU1 expression, which is much lower in cardiac tissue (while MCU and MICU2 are present at similar levels) compared to liver, has been suggested to determine the lower maximal Ca ^2+^ uptake capacity and less-steep Ca ^2+^ dependence that characterise cardiac mitochondria compared to liver mitochondria, as electrophysiological data ^32^ and intracellular Ca ^2+^ measurements ^59^ revealed. The low amount of MICU1 in cardiac mitochondria is instrumental to reduce mitochondrial Ca ^2+^ uptake upon cytosolic Ca ^2+^ elevation, allowing the decoding of the repetitive cytosolic Ca ^2+^ spikes of the beating heart into a graded increase of matrix Ca ^2+^. This integration of frequency fluctuations is crucial to prevent mitochondrial Ca ^2+^ overload, which will be otherwise detrimental to the cardiac myocytes. By contrast, the relatively high MICU1 level and its cooperative activation of MCU in hepatocytes ensure the punctual and massive mitochondrial Ca ^2+^ increase at every single cytosolic Ca ^2+^ spike ^59^, which is functional to liver oxidative metabolism.

An additional degree of complexity came with the discovery of an alternative MICU1 isoform, extremely conserved and expressed at the highest level in skeletal muscle, which originates from the alternative splicing of the MICU1 mRNA, the MICU1.1 splice variant ^60^ (see Figure 1). This alternative isoform was shown to dimerise with MICU2, showing gatekeeper activity similar to that of MICU1, but it binds Ca ^2+^ one order of magnitude more efficiently than MICU1 and activates MCU at lower [Ca ^2+^] _i_ than MICU1 ^60^. Thus, in skeletal muscle, the MICU1.1–MICU2 dimer ensures a much higher net Ca ^2+ ^entry and elevated ATP production than the MICU1–MICU2 dimer ^60^. These results highlight a novel mechanism of the molecular plasticity of the MCU Ca ^2+^ uptake machinery that allows skeletal muscle mitochondria to adapt to the intense metabolic challenge that characterises this tissue (see Figure 2).

### The essential mitochondrial Ca ^2+^ uniporter regulator EMRE

To add further complexity to the study of MCU channel regulation, other molecules have been shown to interact with MCU in recent years. Indeed, the quantitative mass spectrometry analysis of the MCU-interacting proteins revealed the presence of a critical component of the MCU complex, the essential MCU regulator (EMRE) (see Figure 1), a 10 kDa single-pass transmembrane protein that was proposed to be necessary for MCU function by bridging MCU interaction with MICU1 in mammalian cells ^61^. Interestingly, EMRE appears to be required for MCU channel activity in metazoans only, since it is not found in plants, fungi, or protozoa, such as *Dictyostelium discoideum*, although they express MCU proteins with functional channel activity. While its exact role in the MCU complex is still uncertain, EMRE was proposed to mediate MCU sensitivity to matrix [Ca ^2+^] _mt_, suggesting that MCU activity may be modulated by Ca ^2+ ^sensors facing both the IMS (MICUs) and the matrix (EMRE) ^62^. However, this hypothesis was subsequently questioned by other groups, whose data supported an opposite topology for the EMRE protein, with the short N-terminus exposed to the matrix while its acidic C-terminus faces the IMS ^63, 64^. In this conformation, EMRE action would be that of supporting MCU Ca ^2+^ transport activity by interacting with the first TMH of MCU within the IMM and to interact also with MICU1 at the IMS via its C-terminus poly-aspartate tail, thus ensuring MICU1 binding to the channel and gatekeeping activity ^63^.

Recently, the control of EMRE protein levels has emerged as a crucial aspect of MCU complex regulation. Indeed, a correct amount of EMRE is fundamental to guarantee the exact stoichiometry of the different MCU components. Alteration of EMRE protein levels by ablation of the AAA-proteases responsible for its degradation ^65^, or by expression of proteolytic-resistant EMRE constructs ^62^, leads to uncontrolled MCU channel opening causing mitochondrial Ca ^2+^ leakage and mitochondrial Ca ^2+^ overload, resulting in neuronal cell death ^65, 66^. The existence of a tight control of EMRE protein levels has also been demonstrated *in vivo* in the *MICU1 ^–/–^* mice, where EMRE is reduced in a genetic condition characterised by increased [Ca ^2+^] _mt_ basal levels ^48^. These findings clearly underline a negative regulatory mechanism that keeps EMRE expression in check in order to cope with changes in MCU activity ^48^.

### The mitochondrial Ca ^2+^ uniporter regulator 1 MCUR1

Another protein that has been associated with the MCU complex ^67, 68^, despite the original proteomic analysis of the MCU interactors failed to recover it ^61^, is the MCU regulator 1 (MCUR1). MCUR1 is a coiled-coil-containing protein encoded by the *CCDC90A* gene initially identified by a RNAi screen searching for a mitochondrial membrane protein involved in MCU homeostasis ^67^, whose precise function in the regulation of MCU activity and mitochondrial metabolism is still debated. Indeed, MCUR1 has been shown to be required for MCU-dependent Ca ^2+ ^uptake, since its knockdown dampens mitochondrial Ca ^2+ ^entry while its overexpression enhances it ^67^. However, a direct action of MCUR1 on MCU activity was questioned by the finding that its silencing leads to a significant loss of mitochondrial membrane potential (ΔΨ _m_), which *per se* can account for the decreased mitochondrial Ca ^2+^ uptake ^69^. In this context, MCUR1 has been suggested to act primarily as a cytochrome c oxidase (COX) assembly factor ^69^, and this concept would also be supported by the fact that a MCUR1 orthologue is present in budding yeast ^70^, which lack MCU, and is required for yeast COX activity and survival in non-fermentable medium ^69^. Nevertheless, the patch clamp data of Ca ^2+^ currents from voltage-clamped mitoplasts point to an effective role for the MCUR1 protein on MCU channel conductance, independently of ΔΨ _m_^71^. In line with this, experiments in *Drosophila* cells, which are resistant to Ca ^2+^-induced mitochondrial permeability transition (MTP) ^72^ and in which no MCUR1 homologs are found, show increased sensitivity to Ca ^2+^-dependent PTP opening when heterologous human MCUR1 is expressed, which is not accompanied by alterations in the rate of mitochondrial Ca ^2+^ uptake ^72^. Conversely, MCUR1 knockdown in mammalian cells renders them resistant to Ca ^2+^ overload, providing protection from cell death ^72^. This led the authors to conclude that MCUR1 regulates the Ca ^2+^ threshold for the MPT and may act as a molecular bridge connecting the MCU channel to the MTP complex. Whether this hypothesis holds true still has to be tested.

More recently, the genetic deletion of *MCUR1* in endothelial and cardiac tissues *in vivo* by mouse conditional knockout models ^68^ points to an involvement of MCUR1 in the assembly and function of the MCU channel. In particular, MCUR1 binding to MCU and EMRE appears to be necessary for MCU oligomerisation and stability, since *MCUR1 ^–/–^* cells will display a significantly decreased amount of MCU-containing high-molecular-weight complexes and reduced rate of mitochondrial Ca ^2+^ uptake ^68^. This, as expected, impinges on cellular energetics, as it is reflected by the dramatic reduction of ATP levels and consequent activation of the pro-survival autophagy pathway in cells depleted of MCUR1 ^72^. Whether the pleiotropic roles for MCUR1 in the regulation of mitochondrial Ca ^2+^ uptake, OXPHOS efficiency, and the apparent discordant phenotypes of MCUR1-depleted cells depend on the intrinsic transcriptional and metabolic differences of different cell types or on the species-specific features of the mitochondrial Ca ^2+^ machinery is still an open question, and further investigation is needed to clarify it.

## The complexity of mitochondrial Ca ^2+^ uptake regulation

The regulation of the mitochondrial Ca ^2+ ^uptake machinery has been revealed to be far more complex than that resulting from just considering the cooperative actions of the different MCU components and their possible combinations. Indeed, the activity of the MCU channel relies on a multifaceted integration of regulatory mechanisms acting on both the MCU pore subunits and its co-regulators. Moreover, these mechanisms have been shown to occur at multiple levels––transcriptional, post-transcriptional, and post-translational––and their systematic identification has started to be accomplished only in recent years.

In neurons, for example, MCU transcription appears to be under the control of activity-dependent cytosolic Ca ^2+^ signalling through the involvement of calmodulin (CaM) and the activation of CaM-kinase (CaMK) ^73^. It has been shown that the immediate-early gene Npas4, a neuronal transcription factor with neuroprotective action against excitotoxicity ^74^, acts directly downstream of the NMDAR signalling and of CaMK to modulate *MCU* transcription ^73^. In another context, chromatin immunoprecipitation and promoter reporter analyses revealed that the Ca ^2+^-regulated transcription factor cyclic adenosine monophosphate response element-binding protein (CREB) directly binds the MCU promoter and stimulates its transcriptional activity in chicken DT40 lymphocyte cells ^75^. The CREB-mediated activity-dependent modulation of MCU expression in response to intracellular Ca ^2+^ mobilisation via IP3R and store-operated Ca ^2+^ entry (SOCE) has also been proven to be crucial to ensure a prompt metabolic flexibility and cell survival in lymphocytes ^75^.

The existence of post-transcriptional regulation of MCU has been initially described in cancer ^76^, where a strong inverse correlation between MCU expression level and the abundance of microRNA (miR)-25 has been reported in both tumour cell lines and colon adenocarcinoma samples ^76^. Indeed, MCU expression is found to be upregulated in both colon and prostate cancer cells that show a reduced level of miR-25, and the overexpression of miR-25 in these cells downregulates MCU and increases cell sensitivity to apoptosis ^76^. Similarly, in breast cancer, the downregulation of miR-340 is correlated with increased MCU expression in highly metastatic cells, while MCU targeting by miR-340 blocks the metabolic shift from OXPHOS to aerobic glycolysis that would otherwise favour cell migration and invasiveness ^77^. In the context of pulmonary arterial hypertension (PAH), in which vascular cells are hyper-proliferative and apoptosis resistant, exhibiting a cancer-like phenotype, the decreased MCU function that underlies the key phenotypic features of PAH (including elevation of [Ca ^2+^] _i_, reduction of [Ca ^2+^] _mt_, mitochondrial fragmentation, and the Warburg phenomenon) has been ascribed to the downregulation of MCU expression by both miR-25 and miR-138 ^78^. The overexpression of MCU or the blocking of the MCU-targeting miRs in PAH cells indeed restores Ca ^2+^ dynamics, mitochondrial metabolism, and cell proliferation to physiological levels ^78^. MiR-25 has also been shown to target and downregulate MCU in the heart, where it is suggested to protect myocytes from oxidative damage by reducing [Ca ^2+^] _mt_ levels ^79^. Another two muscle-specific miRNAs (myomiR), miR-1 and miR-206, have been implicated in the regulation of MCU expression in muscle ^80^, of which miR-1 plays a major role in the context of cardiac development in both mice and humans. In mice, miR-1 upregulation is crucial during the first few postnatal weeks for the modulation of MCU activity in order to prevent massive [Ca ^2+^] _mt_ elevation that would otherwise occur, since mitochondria distribution undergoes heavy remodelling at this time point and organelles are placed in close contact with the SR Ca ^2+^-releasing channels ^80^.

Post-translational modifications of MCU proteins have also been described. Indeed, the Ca ^2+^-dependent tyrosine kinase Pyk2 has been shown to directly interact and phosphorylate MCU in cardiac cells in response to α-adrenergic stimulation ^81^. The MCU phosphorylation by Pyk2 has been proposed to promote its oligomerisation and to enhance mitochondrial Ca ^2+^ entry, thus stimulating mitochondrial metabolism and favouring cytosolic Ca ^2+^ clearance in cardiac cells ^81^. In addition, the atomic resolution data of the N-terminus of MCU (residues 72–189) allowed the description of the exact structure of the MCU matrix domain ^82^. This domain was shown to acquire a β-grasp-like fold and to bind Mg ^2+^ and Ca ^2+ ^through a MCU-regulating acidic patch (MRAP) ^82^. The disruption of cation binding via mutation of the MRAP sequence impairs MCU oligomerisation, reduces basal [Ca ^2+^] _mt_ levels, and leads to significant dampening of mitochondrial Ca ^2+^ uptake after agonist stimulation, highlighting the role of divalent cations in the regulation of MCU activity ^81^. This has been proposed as a possible feedback mechanism of MCU regulation aimed at preventing excessive mitochondrial Ca ^2+^ entry in conditions of elevated [Ca ^2+^] _i_.

Finally, a wide range of chemical modifications have been reported to target the thiol moiety of Cys residues in many proteins, enabling biological switching of structure and reactivity oxidation ^83^. One of these modifications, S-glutathionylation, was recently shown to occur in MCU protein as the result of oxidation at the evolutionarily conserved Cys-97 ^84^. The S-glutathionylation has been demonstrated to promote a MCU conformational change, which increases oligomerisation ^84^. In human pulmonary microvascular endothelial cells (HPMVECs), this enhances MCU activity and promotes increased mitochondrial Ca ^2+^ entry both in basal conditions and upon agonist challenge, causing cellular bioenergetics crisis and higher sensitivity to cell death ^84^.

Overall, the understanding of the mechanisms controlling Ca ^2+^ entry in mitochondria has made a great step forward in the last few years, and new possibilities for effective modulation of MCU activity have been revealed. In this line, very recently, a systematic orthologous interspecies chemical screen based on the combination of an optimised yeast cell system and a mammalian mitochondria-based NCC library drug screen picked out and validated the first MCU-specific inhibitor molecule to be described: mitoxantrone ^85^. Mitoxantrone has been utilised already in clinical practice for its antineoplastic action against non-Hodgkin’s lymphomas and acute myeloid leukaemia ^86^; however, the anti-tumour properties of this drug appear to rely on a different molecular moiety with respect to its anti-MCU activity, thus opening up the possibility for the chemical engineering of new lead compounds to specifically target MCU function. This is of outmost relevance for the design of novel potential therapeutic approaches to pathologies in which mitochondrial Ca ^2+^ signalling dysfunction is involved ^17, 87, 88^, including those characterised by primary MCU dysfunction due to mutations in its components ^50, 52, 53^.

In conclusion, despite many of the molecular mechanisms refining MCU activity in the cell having been described, of which the most relevant are presented in this review, a number of other possibilities for the modulation of uniporter function may exist (at transcriptional and post-transcriptional levels), and additional efforts are needed from the scientific community to fully unravel the complexity of mitochondrial Ca ^2+ ^uptake regulation.

## Conclusions

In the last few years, tremendous advances have been made in defining the molecular identity of the mitochondrial Ca ^2+^ transport machinery. Most of the components of the MCU complex have been identified, and several cellular and *in vivo* models have been generated that helped to define the physiological relevance of mitochondrial Ca ^2+^ uptake. Still, many issues remain obscure and need to be investigated. The entire spectrum of molecular mechanisms by which different cell types finely tune mitochondrial Ca ^2+^ uptake appears to be related not only to the metabolic needs but also to the breadth of cellular activities modulated by organelle Ca ^2+^ levels. We believe that the complete picture will emerge when at least three conceptual mechanisms are fully investigated, i.e. gene expression (the MCU and MICU1 isoforms show significant tissue-specific differences in expression), alternative splicing (as in the case of MICU1.1), and post-transcriptional and post-translational regulation (which, so far, has been only partially explored). We then need to explore the functional interplay between mitochondrial Ca ^2+^ transport and other ion fluxes, such as Na ^+^ and K ^+^, for which complexity and partial redundancy of transport mechanisms have been described but molecular definition still lags behind. Unravelling this cross-talk will greatly enhance our insight into the regulation of mitochondria, these fascinating organelles with pleiotropic effects on a cell’s life and death.

## Abbreviations

[Ca ^2+^] _i_, intracellular Ca ^2+^ concentration; [Ca ^2+^] _mt_, mitochondrial Ca ^2+^ concentration; CaM, calmodulin; CaMK, calmodulin kinase; CREB, cyclic adenosine monophosphate response element-binding protein; COX, cytochrome c oxidase; EMRE, essential mitochondrial Ca ^2+^ uniporter regulator; ER, endoplasmic reticulum; IMM, inner mitochondrial membrane; IMS, intermembrane space; IP3R, inositol trisphosphate receptor; MCU, mitochondrial Ca ^2+^ uniporter; MCUR1, mitochondrial Ca ^2+ ^uniporter regulator 1; MICU, mitochondrial Ca ^2+^ uptake protein; miR, microRNA; MRAP, mitochondrial Ca ^2+^ uniporter-regulating acidic patch; MTP, mitochondrial permeability transition; OXPHOS, oxidative phosphorylation; PAH, pulmonary arterial hypertension; PGC1α4, peroxisome proliferator-activated receptor gamma coactivator 1 alpha 4; PTP, permeability transition pore; SR, sarcoplasmic reticulum; TMH, transmembrane helix

## Author contributions

Giorgia Pallafacchina wrote the original draft, Sofia Zanin drew the figures, and Rosario Rizzuto revised the manuscript.
